# Prospective swallowing motion self-gating: a feasibility study in carotid artery wall MRI using 3D variable-flip-angle TSE

**DOI:** 10.1186/1532-429X-14-S1-O44

**Published:** 2012-02-01

**Authors:** Zhaoyang Fan, Sven Zuehlsdorff, Xin Liu, Debiao Li

**Affiliations:** 1 Biomedical Imaging Research Institute, Cedars-Sinai Medical Center, Los Angeles, CA, USA; 2 Bioengineering, University of California, Los Angeles, CA, USA; 3 Cardiovascular MR R&D, Cardiovascular MR R&D, Siemens Healthcare, Chicago, IL, USA; 4 Paul C. Lauterbur Research Center for Biomedical Imaging, Shenzhen Institutes of Advanced Technology, Chinese Academy of Sciences, Shenzhen, China

## Summary

This preliminary study aimed to validate a self-gating method in combination with 3D TSE acquisition for gating swallowing motion. The effectiveness in reducing swallowing-related motion artifacts was demonstrated on healthy volunteers and patients.

## Background

3D black-blood MRI is a promising imaging modality for carotid artery wall imaging but is inherently susceptible to motion. Previous work shown that swallowing can result in the greatest motion at the carotid bifurcation and its vicinity (1). A self-gating (SG) method in combination with a 3D variable-flip-angle turbo spin-echo sequence, SPACE, has recently been proposed for gating swallowing motion (2). This work aimed to validate the utility of the new technique on healthy volunteers and patients.

## Methods

Imaging was performed on a 3T system (MAGNETOM Trio; Siemens) using a bilateral four-channel phased array carotid coil (Machnet BV). Eight healthy volunteers (3 M, 5 F, age 18-45 years) and 2 patients with carotid atherosclerosis (2 M, 67 and 74 years) were recruited in the study. All healthy subjects underwent the following three oblique coronal scans using the developed sequence: a) imaging without swallowing instructions or SG (“SPACE STL”); b) imaging with swallowing instructions but without SG (“SPACE SWL”); c) imaging with both swallowing instructions and SG ( “SPACE SWL+SG”). In the “SPACE SWL” and “SPACE SWL+SG” scans, subjects were instructed over the intercom to swallow twice at five preset stages, namely the 30th, 50th, 60th, 70th, and 90th TR of the scan ideally free of motion. The two patients underwent only two scans, SPACE without and with SG, without swallowing instructions. The relevant imaging parameters included: echo time (TE)/TR 141 msec/3 R-R intervals, ECG trigger delay 450-650 msec, readouts in the SI direction, voxel size (after interpolation) 0.35x0.36x0.36 mm3, acquisition time 120 TRs per scan in the absence of motion.

## Results

**Figure 1:**
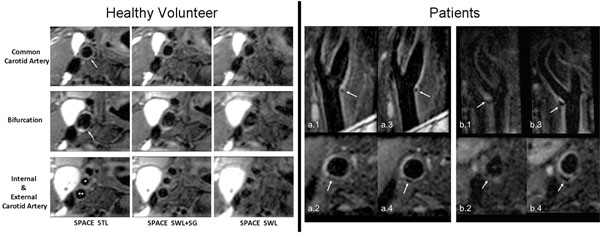
Left panel: Comparison of location-matched longitudinal MPR images obtained from the “SPACE STL”, “SPACE SWL+SG”, and “SPACE SWL” scans, respectively, in a 45-year-old male volunteer. Right panel: Comparison in the depiction of carotid atherosclerotic plaques between regular SPACE (a.1-2, b.1-2) and SG-SPACE (a.3-4, b.3-4) imaging in two patients.

Seven healthy subjects (14 carotids) were included in qualitative and quantitative analyses. In general, swallowing motion resulted in severe blurring of artery wall boundaries, impaired wall continuity, and reduced wall-tissue contrast on non-gated images, which were dramatically improved by means of SG (Fig. 1). Quantitative analysis (image quality scores and wall boundary sharpness) is summarized in Table 1. Regular SPACE imaging provided blurred images in both patients (Fig. 2). In one patient who has random occurrences of shortness of breath, the SPACE images were essentially non-diagnostic. In contrast, the SD-SPACE technique dramatically improved the image quality and the lesions were much better depicted.

**Table 1:** Image quality: 0 (worst) - 4(best) (t-test) and Sharpness (Wilcoxon’s test) obtained from “SPACE STL” are compared with those obtained from two other scans.

	Image quality		Sharpness of carotid wall boundary			
	Score	p-Value	Outer boundary	p-Value	Inner boundary	p-Value

SPACE STL	3.2±0.6	0.002	2.02±0.28	0.232	1.86±0.24	0.121
SPACE SWL+SG	2.4±0.7	N/A	1.95±0.27	N/A	1.78±0.24	N/A
SPACE SWL	1.1±0.5	0.001	1.73±0.37	0.003	1.56±0.36	<0.001

## Conclusions

Swallowing was shown to induce severe overall image degradation, obscure wall boundary, and reduce the wall-to-background contrast. The proposed SG approach significantly mitigated the above problems. Compared with regular SPACE imaging, SG-SPACE provided slightly impaired yet acceptable image quality and statistically comparable wall boundary sharpness in the presence of swallowing events. Considerable improvement has also been observed in two patients. It is anticipated that this approach may greatly enhance the clinical value of 3D black-blood MRI in the assessment of carotid atherosclerosis.

## Funding

NIH 1R01HL096119.

